# How is migration background considered in the treatment and care of people? A comparison of national dementia care guidelines in Europe

**DOI:** 10.1186/s12889-020-09668-4

**Published:** 2020-10-15

**Authors:** Tim Schmachtenberg, Jessica Monsees, Wolfgang Hoffmann, Neeltje van den Berg, Ulrike Stentzel, Jochen René Thyrian

**Affiliations:** 1 German Center for Neurodegenerative Diseases (DZNE), Site Rostock/Greifswald, Ellernholzstraße 1-2, 17489 Greifswald, Germany; 2 University Medicine Greifswald, Institute for Community Medicine, Ellernholzstraße 1-2, 17489 Greifswald, Germany

**Keywords:** Dementia, Migration, Care, Healthcare services, Europe, Guidelines, Policies, Recommendations

## Abstract

**Background:**

People with a migration background are vulnerable to dementia. Due to problems such as underdiagnosis or access barriers, the care of this population is a public health challenge in Europe. Many countries are issuing care guidelines, but a systematic overview of their references to migration groups is lacking. This study aims to analyze national dementia care guidelines regarding their focus on people with a migration background, what specific actions to ensure healthcare have been undertaken at the national level, and whether recommendations for action are made for this population.

**Methods:**

This study is a systematic analysis of national dementia care guidelines of the EU and EFTA (European Free Trade Association) countries. Using the discourse analysis model by Keller (2011), 43 documents from 24 EU and 3 EFTA countries were systematically screened for migration references via keyword and context analysis. The content of the migration-related section was paraphrased, memos and comments were added, and the individual text passages were coded using the strategy of open coding.

**Results:**

Twenty-seven of the 35 EU and EFTA countries have guidelines or similar documents on care for people with dementia, and 12 refer to migration. Norway, Sweden, and Northern Ireland refer to this topic in detail. The focus of the migration-related guidelines is on the early detection and diagnosis of dementia. The main message is that standardized diagnostic tools such as the MMSE (Mini-Mental State Examination) or the clock test are not suitable for linguistic minorities. Nine countries make recommendations for the care of people with a migration background and dementia, but only Norway, Sweden, and Denmark point to available healthcare services. A key recommendation is that the linguistic and cultural background of people should be considered when selecting diagnostic tests. Several countries refer to the validity of the RUDAS (Rowland Universal Dementia Assessment Scale) for migrants.

**Conclusions:**

The topic of migration plays a subordinate role in the dementia care guidelines of European countries. Almost all countries lack appropriate diagnostic tools and healthcare services for people with a migration background. Consequently, this group is vulnerable to underdiagnosis and a lower level of care.

## Background

Caring for people with dementia, especially those who also have a migration background, is a major challenge for public health in Europe. Due to demographic changes, the number of people with dementia in Europe is expected to increase [1] from 9.95 million in 2010 to 13.95 million in 2030 [2]. The prevalence of dementia in people with a migration background (PwM) will increase particularly strongly because the number of older PwM is rising significantly [3] and the risk of dementia increases at a higher age [4]. In the EU, the number of PwM who are over 64 years of age rose from 4.73 million in 2000 to 7.37 million in 2017 [5]. The members of this group are distributed very unevenly across the individual EU countries. More than half of them live in France, Germany, and the UK. Spain, Poland, Italy, Austria, the Netherlands, and Sweden have also large populations of older PwM (2017) [5].

The older migrant population of most European countries and Europe as a whole is characterized by a high degree of linguistic and cultural diversity. There are considerable differences among the EU countries regarding the internal structure of the migrant population. Many European countries, such as France [6], Germany [7], the UK [8], and Spain [9], have a heterogeneous migrant population with large diversity in terms of countries of origin of the largest migrant groups. A few countries, such as Sweden and Poland, have a slightly more homogeneous migrant population. Many countries of origin have geographical proximity, a related national language, and many cultural similarities with the host country [10, 11].

Limited data are available to validly estimate the number of PwM with dementia in Europe. For example, a recent analysis for Germany estimates their number to be 96,500 [12], indicating the need to further examine this group.

Another major problem is the lack of a common definition of migration background at the European level. While the United Nations defines PwM as people who are living in countries other than their country of birth, in Germany, the concept of migration background is based on one’s own and their parents’ citizenship (PwM are not born with German citizenship or have at least one parent who was not born with German citizenship) [13].

There is some evidence indicating that PwM are a vulnerable group in terms of dementia diagnosis and healthcare. Various barriers to seeking help, such as different views and perceptions regarding dementia and care among people with different cultural backgrounds, lack of familiarity of PwM with the respective health care system, stigmatization, and discrimination, are apparent. With respect to stigmatization, the World Alzheimer Report 2019 has described significant country-specific differences. For example, according to the ADI (Alzheimer Disease International) global survey on attitudes toward dementia, 69% of Romanians consider people with dementia to be dangerous, while this figure is 29% in Greece, 20% in Poland, 15% in Germany, and 2 % in Portugal. Almost 67% of the participants from Russia and approximately 58% of Polish people said they wanted to keep dementia a secret. This was the intention of approximately 20% of respondents in Germany and only approximately 3 % of participants in Iran and Kenya [14]. These and other identified country-specific differences in perceptions of dementia are likely to have an impact on the care requirements and utilization of care services by people who emigrated from these countries. Lack of information about the healthcare system and lack of knowledge about existing healthcare services are also often an obstacle to formal care. The organization of healthcare systems and the healthcare concepts of the countries of origin and the host countries often differ considerably [15, 16]. The lack of familiarity with the healthcare system, different perceptions, and stigmatization, together with other factors (convictions and beliefs about dementia (e.g., in some ethnic groups, the widespread perception that dementia is contagious or due to spiritual forces or punishment from God), cultural and language barriers, and inappropriate services [3, 17, 18]), result in underdiagnosis of dementia in PwM [19] and lower use of dementia-related healthcare services among this group [20–24]. Studies have shown that due to the migration background, the validity of the dementia diagnosis is often less accurate and reliable among PwM than in the general population [19]. Thus, different cultural backgrounds must be taken into account to avoid the risk that the growing numbers of PwM with dementia are treated insufficiently or do not use adequate health services [3].

PwM with dementia are in most cases, and even more frequently than people with dementia without a migration background, cared for at home by family members. In many migrant communities, the care of older relatives, who previously cared for them, is a cultural, religious, and emotional norm [25]. The norm of the family-oriented model can lead to exhaustion and stress, especially for female caregivers who do not have the support of an extended family and simultaneously have other important tasks and obligations (job, child care), which can have a negative impact on the care situation. This is particularly the case when care is provided in a country with other cultural norms [26]. Several studies have shown that the psychological burden of family caregivers with a migration background is even higher than for people without a migration background [27–29]. Although there is a great need [30], family caregivers with migration backgrounds take advantage of fewer support services than the majority population [31, 32]. A central cause could be a lack of language and culturally sensitive support services for family caregivers with migration backgrounds or a lack of information about these services [30, 33].

There are efforts in different countries to remedy these problems. On the European level, Alzheimer Europe provides information on established initiatives and materials for the care of PwM with dementia [34]. In the UK, a project exists for people with dementia and their caregivers from black, Asian, and ethnic minorities (The Dementia Alliance for Culture and Ethnicity) [35]. There is also the ETNIMU initiative in Finland for people from the Roma population and people with a Russian, Estonian, or Somali background [36]. Switzerland has initiated the project ‘Doppelt fremd’, which refers to Italian migrants [37]. However, a Europe-wide analysis of existing strategies is lacking.

On the broader level of dementia care in general, several European countries have issued dementia strategies or national dementia plans (NDPs) [38]. While these plans are targeted at the population as a whole, the topic of migration plays a subordinate role in most NDPs [17, 39]. Only 10 of the 35 EU and EFTA countries (28%) have issued an NDP that refers to migration, and one single NDP (the Austrian Dementia Report) contains a separate chapter addressing this topic. Eight NDPs have planned actions to improve care for PwM with dementia, but specific healthcare services for this population exist only in Norway, Northern Ireland, and the Netherlands. Almost all European countries seem to have large gaps in care provision to PwM with dementia on the national level [39]. However, in treatment and care, there are other forms of national guidelines, policies, and recommendations issued by professional medical and nursing associations and health care organizations. Their aim is usually to guide, standardize, and/or increase the quality of treatment and care delivery. A large number of such documents exist, but to our knowledge, there is no systematic analysis of their consideration of migrant-related characteristics. A systematic overview could identify common topics and provide information about approaches in different parts of Europe from which other countries could benefit.

This study aims to analyze national treatment and care guidelines, policies, and recommendations on dementia with regard to PwM. The topics of interest are the guidelines’ focus, what specific actions to ensure healthcare have been undertaken and to what extent at the national level, and whether recommendations for action are made specifically for this target group.

## Methods

This study is a systematic analysis of the public national (political and medical) discourse on the care of PwM with dementia in EU and EFTA countries, represented by written statements in national documents on dementia care.

### Data Sources

In this study, healthcare services at the national level are defined as all services involving healthcare, such as information, support, advice, diagnosis, or treatment plans, which are not limited to specific regions, companies or institutions and are referred to in official national documents by country representatives (e.g., representatives of health ministries, other members of government or representatives of national professional societies).

The following organizations were contacted for information about the existence of national guidelines, policies, and recommendations: national Alzheimer societies (*n* = 28), national health or social ministries (*n* = 32), and national professional societies for geriatrics, gerontology or neurology (*n* = 27) of 31 EU and four EFTA countries. The Alzheimer societies were contacted first (on 02 and 03 May 2019), the health ministries second (on 20 and 21 May 2019), and the professional societies third (on 10 and 11 July 2019). These organizations were asked whether care or treatment guidelines for people with dementia exist at the national level and how these documents could be accessed. The response rate was just over 39% (33 of 87 organizations responded). It was particularly high in the national health or social ministries (almost 72% (23 of 32)) but significantly lower in the national Alzheimer societies (approximately 21% (6 of 28)) and the national professional societies for geriatrics, gerontology or neurology (almost 15% (4 of 27)). The ministries and professional societies were identified by a Google search, while the Alzheimer Europe website served as the basis for the contact data of the Alzheimer societies [40]. In the case of nonresponse by the three organizations contacted, a Google search was carried out to find research institutions, university faculties, medical facilities, clinics, or NGOs, and a PubMed search was conducted to find researchers dealing with the topic of dementia in the individual countries, who were then written to. In two cases (Slovakia and Poland), the respective embassies in Germany and the German embassies in the respective capital were also contacted. Finally, we received responses from 47 organizations of 35 countries and were thus able to perform an analysis for each EU and EFTA country. The list of responding organizations is attached in the appendix (Table 3). To integrate documents from as many countries as possible, no definitions or restrictions were made. All documents mentioned by these organizations were included in this study. The organizations either sent the documents themselves or referred to online platforms where they were accessible. Accordingly, the websites of the national Alzheimer societies, the health ministries, and various professional societies (geriatrics, neurology, psychology) and associations (medical association) served as sources of data. In addition, a Google search was conducted. The corpus of documents for this study was 43 documents. These documents were published in the EU and EFTA countries listed in Table 2. The distribution of the 43 documents among these countries is shown in Table 1.

**Table 1:** Structure of document corpus and overview of publishers

Countries	National document available	Number of documents	Document type by country definition	Document type by own definition	Reason for classification	Publisher
Austria	Yes	2	Guideline Evidence report	Guideline Evidence report	Evidence-based Nonbinding No recommendations	Competence Center Integrated Care, Vienna Regional Health Insurance Fund Ministry of Health
Belgium (Flanders)	Yes	4	Memorandum Memorandum Action framework Transition plan	Recommendations Recommendations Guideline Guideline	Low normative character Evidence-based Nonbinding Evidence-based Nonbinding	Dementia Competence Center, Alzheimer League European Patent Office publishing company Cabinet Minister for Public Health
Bulgaria	Yes	1	National consensus	Guideline	Evidence-based Nonbinding	Society of Neurology
Croatia	No	0				
Cyprus	No	0				
Czech Republic	Yes	3	Recommendations (three times)	Recommendations (three times)	Evidence-based Nonbinding	2 x Neurological Clinic, 1 x Society for General Practitioners
Denmark	Yes	4	Policy (four times)	Guideline (four times)	Evidence-based Nonbinding	Ministry of Health
England, Wales	Yes	1	Policy	Guideline	Evidence-based Nonbinding	NICE – Institution of the Ministry of Health
Estonia	Yes	1	Guideline	Guideline	Evidence-based Nonbinding	Society for Neurologists and Neurosurgeons
Finland	Yes	1	Recommendations	Guideline	Evidence-based Nonbinding	Medical Council
France	Yes	4	Guideline Continuation sheet Recommendations Recommendations	Guideline Continuation sheet Recommendations Recommendations	Evidence-based Recommendations Low normative character	Ministry of Health Ministry of Health Ministry of Health Ministry of Health
Germany	Yes	1	Guideline	Guideline	Evidence-based Nonbinding	Society of Neurology, DGPPN
Greece	No	0				
Hungary	Yes	1	Protocol	Guideline	Evidence-based Nonbinding	Ministry of Health
Ireland	Yes	1	Guideline	Guideline	Evidence-based Nonbinding	Quality and Safety in Practice Committee
Italy	No	0				
Latvia	Yes	1	Policies	Guideline	Evidence-based Nonbinding	Society for Neurodegenerative Diseases
Lithuania	No	0				
Luxembourg	Yes	1	Guideline	Recommendations	Low normative character	Ministry of Health
Malta	Yes	1	–	Recommendations	Low normative character	Ministry of Health
Netherlands	Yes	2	Policy Guideline	Guideline Guideline	Systematically developed Evidence-based Nonbinding	Association for Clinical Geriatrics Ministry of Health, Alzheimer Netherlands
Northern Ireland	Yes	1	Policy	Guideline	Evidence-based Nonbinding	The British Psychological Society and Gaskell
Poland	No	0				
Portugal	Yes	1	Standards	Guideline	Evidence-based Nonbinding	Ministry of Health
Romania	Yes	1	Guideline	Guideline	Evidence-based Nonbinding	Ministry of Health
Scotland	Yes	1	Standards	Policy	Instruction, legal foundation	Government
Slovakia	No	0				
Slovenia	Yes	2	Policy Official gazette	Guideline Strategy	Evidence-based Nonbinding No recommendations	Psychiatric Association of the Medical Council Government
Spain	Yes	1	Guideline	Guideline	Evidence-based Nonbinding	Ministry of Science and Innovation
Sweden	Yes	2	Policy (two times)	Guideline (two times)	Evidence-based Nonbinding	Central Office for Health (governmental authority) Dementia diagnosis
Iceland	Yes	1	Guideline	Guideline	Evidence-based Nonbinding	Ministry of Health
Liechtenstein	No	0				
Norway	Yes	1	Policy	Policy	Legal basis	Ministry of Health
Switzerland	Yes	3	Policy Recommendations Recommendations	Policy Guideline Recommendations	Legally binding Evidence-based Low normative character	Academy of Medical Sciences Medical associations Swiss Alzheimer’s Association
**EU/EFTA**	**27**	**43**				

*DGPPN* German Society of Psychiatry and Psychotherapy, Psychosomatics and Neurology

### Procedures

The documents were heterogeneous and contained different document types with different definitions of policies, guidelines, and recommendations (Table 1). To structure this corpus, the documents were assigned to the following standardized categories.

#### Policies

Instructions for action published by legally legitimated institutions that must be followed in a binding manner and that reflect the state of knowledge of medical science at a certain point in time [41–43].

#### Guidelines

Systematically developed and scientifically based, legally nonbinding decision-making assistance on the appropriate procedure for specific health problems [44, 45].

#### Recommendations

Suggestions, advice, hints, or consensual solution strategies for selected questions. Recommendations have lower scientific evidence and a lower normative character than guidelines [42, 43].

Subsequently, the content of the documents was described. First, the tables of contents were examined for an existing migration chapter. Then, the continuous text was screened for the following key terms: minorities, minority, migration, culture, ethnic, background, migrant, sensitive, cultural, diverse, diversity, language, origin, nonwestern, characteristic, communities, religious, native and guest. If a migration reference could be identified, the content of the respective section was subjected to a detailed analysis. The fine analysis was based on Keller’s model of qualitative discourse analysis (2011). This knowledge-sociological approach aims to reveal the processes and practices of knowledge production at the level of institutional fields. This method can be used to reconstruct whether and to what extent discourses establish or organize relations between phenomena. Thus, this model is a suitable approach to reveal the extent to which the relationship between dementia and migration is considered in official documents at the national level and what knowledge about the care situation of PwM with dementia exists or is communicated [46].

The data were analyzed according to the following scheme: 1. the relevant text passages were read repeatedly; 2. the contents were paraphrased; 3. the individual text passages were assigned memos and comments; 4. the text passages were coded; 5. the statement contents were recorded and reconstructed in an interpretative-analytical way; 6. the empirical results were interpreted and assessed; 7. the results were presented in tabular and text form. The comments described which criteria were used to formulate the respective codes and assign them to a text passage, and the memos documented what further considerations and hypotheses arose regarding the specific text passage. For the coding of the text passages, the strategy of open coding was used. The categories were derived from the contents of the texts [46]. Table 2 shows the categories derived from the documents that were analyzed. The data coding was carried out by the first author. In this study, the data were first interpreted individually for each country, then short country profiles were produced, and finally, the findings were compared.

**Table 2:** Reference of the national dementia care guidelines of the EU/EFTA countries to migration

Countries	Migration reference of national guidelines		Subthemes related to migration								Migrant-related needs, services, and recommendations for action		
	Reference to migration	Chapter on migration	Needs	Dementia diagnosis	Care	Care-inequalities	Service access	Utilization of formal services	Care barriers	Suitability screening tests	Identification of special needs	Specific services available	Recommendations for action
Norway	Х	Х	Х	Х	Х	Х	Х	Х	Х	Х	Х	Х	Х
Sweden	Х	Х	Х	Х	Х	Х	Х	Х	Х	Х	Х	Х	Х
Northern Ireland	Х	–	Х	Х	Х	Х	Х	Х	Х	Х	Х	–	Х
Spain	Х	–	Х	Х	Х	Х	–	Х	Х	Х	Х	–	Х
Scotland	Х	–	Х	Х	Х	Х	Х	–	Х	–	Х	–	Х
Belgium (Flanders)	Х	–	Х	–	Х	Х	Х	–	Х	–	Х	–	Х
England, Wales	Х	–	–	Х	Х	Х	Х	–	–	Х	–	–	Х
Denmark	Х	–	Х	Х	–	–	–	–	–	Х	Х	Х	–
Germany	Х	–	–	Х	–	–	–	–	Х	–	–	–	Х
Austria	Х	–	–	Х	–	–	–	–	–	Х	–	–	–
Ireland	Х	–	–	Х	–	–	–	–	–	Х	–	–	–

### Language of national dementia care guidelines

The country-specific institutions and experts were mainly contacted in English, while the German-speaking countries were contacted in German. In some (mainly Eastern European) countries, follow-up contact was made in the respective national language. For this purpose, the translation program DeepL and Google Translator were used. The 43 documents sent in by the institutions and experts were mostly (28) written in the respective national language. Eight documents (1 each from England, Wales, Scotland, Ireland, Malta, Flanders, Spain, and the Netherlands) were available in English and 7 documents (3 from Switzerland, 2 from Austria, 1 each from Germany and Luxembourg) in German. Of the 28 documents published exclusively in the respective mother tongue, 9 (4 from France, 3 from Belgium/Flanders, 1 each from the Netherlands and Portugal) were translated with the help of DeepL. The remaining 19 documents were searched for keywords in the respective national language following a Google search and with the help of Google Translator. To ensure the rigor of the study, a workshop was organized in The Hague (Netherlands) on 22 October 2019 with experts from various EU and EFTA countries, where the results of this analysis were discussed.

## Results

In 24 of 31 EU countries (77.5%) and three of four EFTA countries, there are documents at the national level with recommendations, guidelines, or policies for the care of people with dementia. The 27 EU and EFTA countries provided a total of 43 documents. Most of these are guidelines (30). Only three countries (Scotland, Norway, and Switzerland) have policies. In addition, seven recommendations for action and three reports/strategies were taken into account. Eight countries (Greece, Italy, Croatia, Liechtenstein, Lithuania, Cyprus, Slovakia, and Poland) have no such documents (Table 1). Fifteen documents from 11 EU countries (Belgium/Flanders, Denmark, Germany, England, Ireland, Northern Ireland, Austria, Scotland, Sweden, Spain, and Wales) and the EFTA country Norway consider the topic of migration. Twenty-eight documents from 13 EU and two EFTA countries do not refer to migration. Documents from Norway and Sweden have a chapter on migration (Table 2). Most other countries refer only briefly, with individual sentences or short sections, to specific aspects of this topic. In addition to country-specific differences, there are document type-specific differences. While none of the three reports/strategies refers to migration, two of seven recommendations, 11 of 30 guidelines, and two of three policies have a reference.

### Overview of country-specific guidelines

This section presents the results for the individual countries that have national guidelines with a migration reference. The results are structured according to the respective themes and subthemes and are presented descriptively. In Table 4 attached in the appendix, supporting quoted text excerpts are presented.

#### Austria

Document: “Medical guideline for integrated care for dementia patients” from 2011. Theme: Dementia diagnosis; key message: Neuropsychological tests for differential diagnosis must take into account a person’s sociocultural background and language skills. Subtheme: Diagnostic tools; key message: The significance of the Mini-Cog-Screening-Test is not affected by linguistic and cultural differences [47].

#### Belgium (Flanders)

Document 1: “Memorandum” from 2019. Theme: Population of older migrants; subtheme: Development; key message: The number of older people with an Italian, Moroccan, or Turkish background is increasing [48]. Document 2: “You and me, together we are human: a reference framework for quality of life, housing and care for people with dementia” from 2018. Theme: Challenges of diversity for healthcare; key message 1: The increasing diversity in Western societies poses challenges for caregivers. Key message 2: Cultural and ethnic background affects the view of dementia and which aspects of care are considered important. Key message 3: In some cultures, dementia is strongly tabooed. Subtheme: Recommendations; key message 1: People from these cultures need to be better informed, and their awareness of dementia should be increased. Key message 2: Care facilities should take into account the culture-specific needs of PwM without falling into stereotyping and overculturalization [49].

#### Denmark

Documents: “National clinical guidelines on the examination and treatment of dementia” from 2013, guidelines on the “diagnosis of mild cognitive impairment and dementia” from 2018. Theme: Population of older migrants; subtheme: Development; key message: The number of older people from non-Western countries is increasing. Theme: Dementia diagnosis; subtheme: Challenges regarding people from certain ethnic groups; key message: The diagnosis of dementia among people from certain ethnic groups is complicated by linguistic and cultural differences [50, 51]. Subtheme: Diagnostic tools: The screening tool RUDAS (Rowland Universal Dementia Assessment Scale) has been validated for PwM [51].

#### England and Wales

Document: NICE Guideline “Dementia: Assessment, management and support for people living with dementia and their carers” from 2018. Theme: Service access; key-message: People from minority ethnic groups have less access to health and social services. Subtheme: Recommendations; key message: Service providers should design their services to be accessible to people from ethnic minorities. Theme: Dementia diagnosis; subtheme: Diagnostic tools; key message: Some diagnostic tools are not appropriate for cultural differences and language deficits, leading to biased outcomes among certain population groups. Sub-subtheme: Recommendations; key massage: Health and social service providers are recommended to consider the appropriateness of cultural and linguistic differences when selecting diagnostic tests [52].

#### Germany

Document: “S-3 guideline Dementias” from 2016. Theme: Dementia diagnosis; subtheme: Diagnostic tools; key message: A person’s sociocultural background and language competence can influence the results of neuropsychological procedures in the diagnosis of dementia. Sub-subtheme: Recommendations; key message: Neuropsychological tests for differential diagnosis of questionable or mild dementia must take into account a person’s sociocultural background and language skills [53].

#### Ireland

Document: “Dementia: Diagnosis & Management in General Practice” from 2019. Theme: Dementia diagnosis; subtheme: Diagnostic tools; key messages: A person’s cultural background can affect her performance in cognitive impairment screening tests. Two instruments are mentioned that are particularly appropriate for ethnic minorities: The MIS (Memory Impairment Screen) and the Mini-Cog Screening Test [54].

#### Northern Ireland

Document: “Dementia: A NICE–SCIE Guideline on supporting people with dementia and their carers in health and social care” from 2007. Theme: Care; subtheme: Needs; key-message: People from black and ethnic minority communities have special linguistic, cultural, religious, and spiritual needs. Theme: Dementia diagnosis and care; subtheme: Access; key message: Ethnic minorities are a risk group for underdiagnosis and a lower level of care. Sub-subtheme: Causes; key message: Communication difficulties, culturally and linguistically inadequate care, stigmatization, family pressure, and a lack of knowledge about care options are causes for this. Theme: Development and effects of dementia; subtheme: Vulnerability; key message: Nonnative English-speaking people are vulnerable to the effects of dementia, and older people from Africa, the Caribbean, and Asia are a risk group for developing vascular dementia. Theme: Healthcare services; subtheme: Recommendations; key message: Care providers should develop special support services, special information material, and culturally oriented training for ethnic minorities. Theme: Dementia diagnosis; subtheme: Diagnostic tools; sub-subtheme: Recommendations; key-message: With regard to dementia screening tests for nonnative speakers and language barriers, the use of independent interpreters and the provision of information in the preferred language are recommended [55].

#### Norway

Document: “National professional guidelines on dementia” from 2017. Theme: Care; subtheme: Needs; key message: People with minority backgrounds have special needs (other ideals, ideas, and desires regarding information and self-determination). Theme: Dementia diagnosis and care; subtheme: Validity and access; key message: People with minority backgrounds are vulnerable to misdiagnosis of dementia and lower utilization of healthcare services. Sub-subtheme: Causes; key messages: Their cultural and linguistic background can complicate the investigation. The cognitive tests used are not suitable as assessment tools for people from different immigrant groups. Theme: Dementia diagnosis; subtheme: Diagnostic tools; sub-subtheme: Recommendations; key message: The use of the intercultural screening test RUDAS is recommended for people with a different cultural and language background as well as an extended assessment by the specialist medical service and a neuropsychological examination. Theme: Healthcare services; subtheme: Information; sub-subtheme: Availability; key messages: The Ministry of Health and the Competence Center for Migration and Minority Health (NAKMI) has published information on dementia in four different languages (Norwegian, English, Polish, and Urdu) as well as a brochure on interpreters in the health system [56]. This brochure provides information on the tasks, requirements, and guidelines for professional interpreters and gives an overview of the rights, duties, and information/compliance bodies for people who use the services of an interpreter [57].

#### Scotland

Document: “Standards of Care for Dementia in Scotland” from 2011. Theme: Dementia treatment; subtheme: Communication; key message: Language, cultural, and ethnic barriers pose a challenge to communication in dementia treatment. Theme: Dementia diagnosis; sub-theme: Recommendations; key message: Scotland’s National Health Service Boards should ensure that people with dementia from black and ethnic minority communities are given timely access to the diagnosis of dementia. Theme: Healthcare services; subtheme: communication and language support; sub-subtheme: Recommendations; key message: Communication and language support should be offered in the case of language, cultural, and knowledge barriers [58].

#### Spain

Document: “Clinical Practice Guideline on the Comprehensive Care of People with Alzheimer’s Disease and other Dementias” from 2010. Theme: Care; subtheme: Impact of culture and language; key message: Cultural and language elements have an impact on diagnosis, opportunities for health and social care, participation in support services, and the risk of abuse related to dementia. Theme: Care; subtheme: Cultural background of caregivers; key message: Dementia care is increasingly provided by caregivers with migration backgrounds (especially by young immigrants from Latin America). Theme: Healthcare services; subtheme: Information and communication; sub-subtheme: Recommendations; key message: Individual information services with consideration of culture, religion, and ethnic origin should be developed and communication support by a cultural mediator in case of language barriers should be provided [59].

#### Sweden

Document 1: Policy “Health and social care at Dementia” from 2017. Theme: Healthcare; subtheme: Rights of linguistic minorities; key message: Linguistic minorities have the right to individually and linguistically adapted information about health status and available care services. Theme: Care; subtheme: Inpatient care; sub-subtheme: Recommendations; key message: Stationary facilities should design the physical environment of residents with dementia according to their cultural and religious needs [60]. Document 2: 2018 evaluated version of the policy from 2017. Theme: Dementia diagnoses; subtheme: Early detection; sub-subtheme; Access; key message: People with different language or cultural backgrounds have lower chances of early detection. Theme: Healthcare services; subtheme: Availability; key message: There is a lack of appropriate drug treatment and specific care services (daycare, home care, and family care) for this group. Subtheme: Access; key message: People born abroad benefit less from community support than people born in Sweden. Theme: Dementia diagnosis and treatment; subtheme: Recommendations; key message: Districts and municipalities should work more actively to diagnose dementia in people from other countries and to gain more knowledge about the examination and treatment of dementia in this group. Theme: Healthcare services; subtheme: Diagnostic tool; sub-subtheme: Availability; key messages: Sweden has validated the RUDAS assessment tool for linguistic and cultural minorities and developed a training program for the use of this tool. Currently, approximately half of the Swedish districts use RUDAS [61].

### Countries without migration reference in national care guidelines

Bulgaria, the Czech Republic, Estonia, Finland, France, Hungary, Iceland, Latvia, Luxembourg, Malta, the Netherlands, Portugal, Romania, Slovenia, and Switzerland do not refer to migration in their national policies, guidelines or recommendations on the care and treatment of dementia.

### Comparisons between countries

There are clear differences in the scope of the national documents on dementia care of the EU and EFTA countries (Northern Ireland: 392 pages; Norway: 304 pages; Germany: 134 pages; Bulgaria: 32 pages; Iceland: 10 pages). These differences have a significant impact on reference to migration. The documents with a large number of pages (Northern Ireland, Norway) address this topic in detail, those with a medium size (Germany) address it briefly, and those with a small number of pages (Bulgaria, Iceland) do not address it at all. Furthermore, there are significant differences in the publication dates (Hungary: 2005; Northern Ireland: 2007; Luxembourg: 2018; Ireland: 2019). However, no relationship can be identified between the publication date and the migration reference. There are both older documents (Northern Ireland, Spain) that take the topic into account and newer documents (Luxembourg, France) containing no reference. Particularly noticeable in the comparison of the documents are the different names of the people considered PwM in this study (Norway: immigrants, people with minority backgrounds [56]; UK: people from minority ethnic groups [52, 55]; Belgium/Flanders: people with a migrant background [48, 49]; Spain: people from different cultural or religious groups [59]; Sweden: people with different cultural or linguistic backgrounds [60], people born abroad [61]).

The content focus of the sections on the care of PwM with dementia in the migration-related documents is on early detection and diagnosis. Only Belgium (Flanders) does not take this topic into account. The main problem identified is that the cultural background and language skills of PwM can influence the results of dementia diagnostic tests [52–54, 56, 59]. Consequently, the focus in most countries (9 out of 12) is on the suitability of cognitive screening tools for minority groups. Norway, Northern Ireland, England, Wales, and Spain report that standardized cognitive tests such as the MMSE or the clock test are not suitable for people with a different linguistic or cultural background [52, 55, 56, 59]. Ireland and Austria refer to cognitive screening tests such as the MIS and the Mini-Cog Screening Test, which are less prone to linguistic and cultural influences [47, 54]. Norway, Sweden, and Denmark point to the validity of RUDAS for people with a different linguistic or cultural background [51, 56, 60]. The second central topic is the existence of care inequalities between ethnic minorities and the majority population (in 8 of 12 countries). Norway and Sweden note that PwM use fewer formal healthcare services (primary healthcare services, community support services, inpatient care services) [56, 61]. In seven countries, the access of PwM with dementia to healthcare services is discussed. Some countries report that PwM or ethnic minorities have less access to adequate healthcare services [52, 56], and they have lower chances of early detection and appropriate drug treatment [61]. Six countries point to care barriers such as stereotyping or linguistic, cultural, and ethnic barriers. As a result, PwM are mentioned by several countries as a risk group for underdiagnosis and lower use of care [52, 55, 61]. Seven countries identify that PwM with dementia have special needs. They refer to a different perspective on dementia, different preferences for care, and other ideals, ideas, and desires regarding information and self-determination [55, 56, 59, 60].

**Fig. 1:**
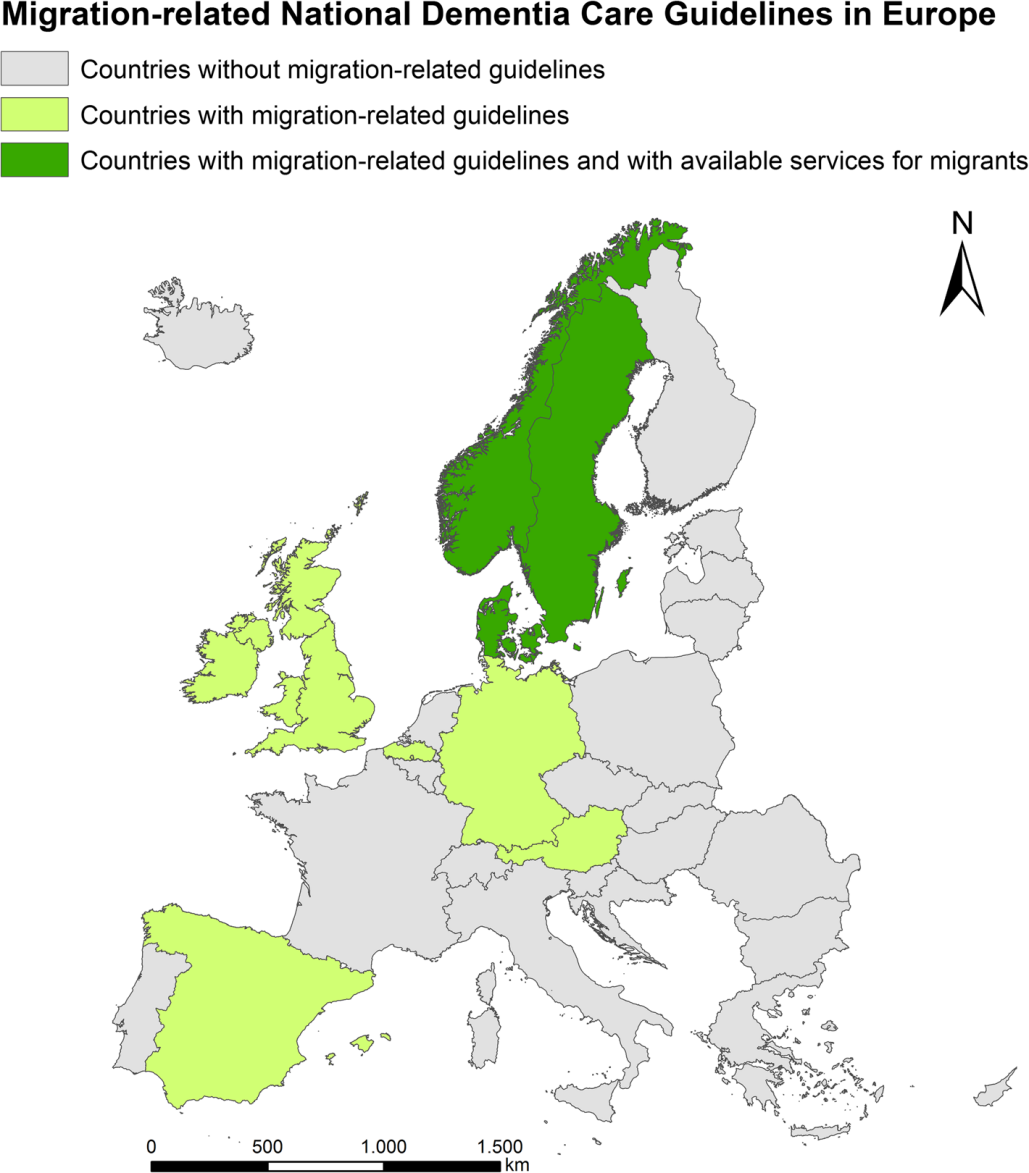
EU/EFTA countries with migration-related National Dementia Care Guidelines and available healthcare services (as of 11.07.2019) (source of the map in Fig. 1: The map was created by the authors with the software ESRI®ArcGIS™ 10.5.1, Esri Inc., Redlands, California (USA), for the use of which a license was required. Geo data source: European Commission, Eurostat (ESTAT), GISCO)

Nine countries provide recommendations for the care of PwM with dementia. Norway, Sweden, Germany, England, and Wales recommend that the linguistic and cultural background of people should be taken into account when selecting diagnostic test procedures [52, 53, 56, 61]. Norway, Sweden, Northern Ireland, and Spain recommend that care providers offer specific support and information to people with dementia and their ethnic minority relatives, taking into account their cultural, religious, and linguistic needs [55, 56, 59, 60]. Norway, Northern Ireland, and Spain note that information in the preferred language and an independent interpreter should be offered to people with dementia and their caregivers in case of language barriers [55, 56, 59]. Currently, only Norway, Sweden, and Denmark have specific healthcare services at the national level for PwM with dementia (Fig. 1). Norway has published informational material on dementia in four different languages (Norwegian, English, Polish, and Urdu) and a brochure with information on rights, requirements, and guidelines concerning the provision and use of professional interpretation services [56]. Sweden has adapted RUDAS to people with different linguistic and cultural backgrounds and developed a training program for health professionals regarding the application of this tool [61]. Denmark has validated RUDAS for PwM [51]. Sweden, Denmark, England, Wales, and Belgium (Flanders) follow an integrative care model. They adapt the mainstream services of the healthcare system to people with different linguistic or cultural backgrounds [49, 51, 52, 60, 61]. Northern Ireland recommends that healthcare providers develop specialized services for ethnic minorities [55]. The Norwegian Directive pursues a segregative care strategy with specialized services for cognitive assessment, dementia diagnosis, and follow-up, while subsequent treatment and care are provided as part of general medical care [56]. This study has shown that some models of good practice exist in individual countries, but in Europe, as a whole, there is a significant gap in care for PwM with dementia.

### Relationship between population size of older migrants in individual countries and migration reference in national dementia care guidelines

According to this analysis, there is a relationship between the absolute size of the population of PwM who are at an age that is associated with a higher risk of dementia (65 years or older) and the consideration of migration in dementia care guidelines. If countries with a large older migrant population (over 200,000) publish national guidelines on dementia care, the likelihood of a migration reference is much higher than if countries with a small migrant population (under 100,000) publish such documents. The example of France (largest older migrant population, no migration-related guidelines) shows that a large older migrant population does not automatically lead countries to include the topic of migration in national dementia care guidelines [5].

## Discussion

Similar to the study on the focus of NDPs in the care of PwM with dementia [39], this analysis shows that migration plays a subordinate role in national documents on dementia care. More than half of the countries with national guidelines, policies, or recommendations do not refer to this topic. Most of the documents from the 12 countries with a migration reference address it only briefly. There is broad consensus in the migration-related documents that standardized cognitive test procedures are not suitable for linguistic and cultural minorities. The Alzheimer Europe report “The development of intercultural care and support for people with dementia from minority ethnic groups” confirms this finding and concludes that most standardized tools used in European countries to diagnose dementia are not suitable for use with people from ethnic minority groups. According to the report, there is not yet an instrument that is perfectly tailored to the needs of this group. The MMSE, which is one of the most widely used cognitive screening tools in Europe, has a cultural, social, ethnic, and educational bias [3]. In addition, in many countries, reference is made to inequalities in care, such as the lower utilization of formal healthcare services by PwM and the lower offering of appropriate services compared to the majority population. Several studies also report that PwM or people from minority ethnic groups are underrepresented in dementia services [21, 22, 62, 63], and ethnic minority caregivers use fewer formal services than the majority population [31, 32]. Causes discussed are language problems, cultural views on dementia and care, lack of information on available care services, and lack of culturally and linguistically appropriate care services [15, 24, 25, 64, 65]. Furthermore, in several studies, PwM report negative encounters with healthcare providers and experiences of discrimination and racism by health professionals [66–69]. Some of these care barriers are also identified in the national dementia care guidelines analyzed in this study. In most of the guidelines with a migration reference, PwM are identified as a risk group for underdiagnosis and a lower level of care. This result is important as it shows for the first time which national care guidelines of EU and EFTA countries identify the vulnerability of PwM in terms of diagnosis and care. Since the problem identification is the basis for the adoption of measures, policy-makers in dementia care are given an indication of which European countries are expected to focus PwM in future care planning. In this way, possible models of good practice can be identified and transnational networking of care providers can be promoted.

In the care guidelines of countries such as Northern Ireland, which identify PwM or ethnic minorities as a risk group for underdiagnosis or a lower level of care, recommendations are already given for the care of PwM with dementia. A total of nine countries provide such recommendations. They recommend, inter alia, that care providers should provide people with dementia and their relatives from ethnic minorities with specific information and support services that take their cultural and linguistic background into account. Various studies also identify a large need for relief and support services for family caregivers of PwM with dementia, which must be oriented toward the individual and cultural needs of PwM and their relatives [26, 70–74]. In particular, the importance of mother-tongue education about dementia and culturally sensitive treatment and support services [73], for example, through dementia hotlines [26], specific counseling centers, or printed information materials [74], is emphasized. Furthermore, the need for help with physical care activities, support in the household, and, especially, more emotional and mental support is pointed out [70, 71]. Another finding of this study relevant for identifying models of good practice is that only the documents from Norway, Sweden, and Denmark refer to currently available specific healthcare services for PwM with dementia. Norway provides multilingual information material on dementia. Sweden and Denmark have adapted the cognitive screening tool RUDAS to people with different linguistic and cultural backgrounds. Similar to some of the guidelines analyzed [51, 56], RUDAS is recommended in further reports and articles for the diagnosis of dementia in people from ethnic minority groups [3, 26] because the different tests are not influenced by gender, cultural background, and language [3]. This study confirms the findings of the study on NDPs of the EU and EFTA states [39] that in almost all European countries, a lack of culturally and linguistically appropriate healthcare services for PwM with dementia exists at the national level.

In addition, document type-specific differences with regard to migration references become apparent. While none of the reports considers the topic of dementia in PwM, two of seven recommendations, 11 of 30 guidelines and two of three policies refer to this topic. Thus, the proportion of migration-related documents increases with their scientific evidence, normative character, and legal relevance. Furthermore, documents with a large scope refer to migration in more detail than documents with a medium size, while documents with a small scope do not take this topic into account. These findings illustrate that the topic of dementia and migration is not at the top of the political agenda in European states. It is only considered when the documents have a larger scope, stronger scientific input, and greater legal relevance, as illustrated by the fact that most of the policies, but only a quarter of the recommendations, consider migration. The country-specific differences can also be explained by the different thematic focuses of the documents and the different relevance of migration in the individual countries. A further influencing factor may be the different terms used regarding PwM (e.g., Ireland: ethnic minorities [54]; Spain: people from different cultural or religious groups [59]; Sweden: people born abroad [61]). These terms are based on various definitions and a different understanding of constructs such as “ethnicity” [3]. The different use and understanding of terms regarding PwM by different countries and experts represents a major challenge for healthcare policymakers and scientists [75]. For a more exact determination of the importance of the topic of dementia and migration in Europe, a better comparability of country-specific data, and the provision of migrant-specific recommendations for action a uniform use and definition of a term regarding PwM would be essential [3].

Overall, this study provides the first overview of country-specific and transnational guidelines for the care of PwM with dementia. It offers an indication of the countries in which national dementia-specific care strategies have a focus on PwM and in which countries this group does not play a special role in care planning. This information can be used by researchers for further country-specific analyses regarding the focus of care strategies on PwM and by care planners for the initiation of targeted cooperation with care providers from countries that are planning or have already implemented specific measures to care for PwM with dementia.

## Limitations

Except for Belgium/Flanders, this study refers only to national policies, guidelines, and recommendations on dementia care published by national organizations or authorities such as the Alzheimer societies, professional societies, or ministries of health. Therefore, only those documents were taken into account that were sent on request by the respective organizations or ministries. We cannot rule out the possibility that organizations and documents exist that were not identified or contacted by the authors. However, the organizations involved in this study were asked to refer to appropriate information or informants, which we then included. Nevertheless, there were also organizations that did not give any response. Accordingly, in the individual EU and EFTA countries, there are other documents on the care of people with dementia (e.g., at the local level) that were not included in this study. However, this was not the aim of the study, and the inclusion of these documents would have compromised the standardization of the procedure and would have reduced comparability.

In addition, due to the level of comparison (across nations, countries, and languages), there are differences in the definition of the target group (e.g., immigrants, minority ethnic groups) and thus different terms used in the context of migration in the analyzed documents. Furthermore, there is a certain heterogeneity in terms of content focus, aims, scope, publisher, publication dates, and type of documents. These difference limit comparability; however, these limitations are well known in international comparison research and must be weighed against the new knowledge generated. This new knowledge needs to be taken into account in further research.

## Conclusions

This study supplements the existing literature with a systematic analysis of the migration reference in the EU and EFTA countries’ national dementia treatment and care guidelines, policies, and recommendations. The topics of interest were the migration-related content focus, the specific actions taken to ensure healthcare provision and the recommendations made for the care of this vulnerable population. Currently, migration plays a subordinate role in national documents on dementia care. Only 3 of 35 EU and EFTA countries (Norway, Sweden (both with their own chapters), and Northern Ireland) refer in their guidelines, policies, or recommendations in detail to the topic of migration. The focus of the migration-related documents is on early detection and diagnosis of dementia. The main message of these documents is that standardized cognitive test procedures such as the MMSE or the clock test are not suitable for linguistic and cultural minorities. To tackle this problem, several countries recommend that the linguistic and cultural background of PwM should be taken into account when selecting diagnostic test procedures. While Ireland and Austria point out that the cognitive screening tests MIS and Mini-Cog are less prone to linguistic and cultural influences, Norway, Sweden, and Denmark refer to the validity of RUDAS for people with a different linguistic or cultural background. In most countries with migration-related national documents on dementia care, PwM are identified as a risk group for a lower level of care and underdiagnosis. To address this problem, policy-makers, researchers, and care providers should pay more attention to the translation, validation, and nationwide availability of multicultural dementia diagnostic tools such as RUDAS for PwM. In addition, specialized tools for PwM with language, cultural, and/or educational barriers should be developed and tested. We assume that the current lack of migrant-specific diagnostic tools at the national level, if not remedied in a timely manner, may lead to a growing population being excluded from care.

## Data Availability

Most of the data analyzed in this study are available on the websites of the national Alzheimer societies, ministries of health or professional societies (societies of geriatrics, gerontology, neurology) of the EU and EFTA countries or on the search engine Google (Austria: https://www.demenzstrategie.at/fxdata/demenzstrategie/prod/temedia/practicalexamplesdocuments_file/4b_Medizinische_Leitlinie-Besser_leben_mit_Demenz.pdf, Belgium/Flanders: https://wyldementia.org/wp-content/uploads/2018/12/You-and-me-together-we-are-HUMAN-ST.pdf, Denmark: https://www.sst.dk/-/media/Udgivelser/2018/National-klinisk-retningslinje-for-demens-og-medicin.ashx?la=da&hash=457F5983E29B8595262D66EFA91608A55F48BAB2, England/Wales: https://www.nice.org.uk/guidance/ng97/resources/dementia-assessment-management-and-support-for-people-living-with-dementia-and-their-carers-pdf-1837760199109, Germany: https://www.awmf.org/uploads/tx_szleitlinien/038-013l_S3-Demenzen-2016-07.pdf, Ireland: https://www.icgp.ie/speck/properties/asset/asset.cfm?type=LibraryAsset&id=7A86D043%2DCFD5%2D4008%2D834C08BC9E6EFC74&property=asset&revision=tip&disposition=inline&app=icgp&filename=Dementia%5FQRG%5F15th%5FApril%5F2019%2Epdf, Northern Ireland: https://www.scie.org.uk/publications/misc/dementia/dementia-fullguideline.pdf?res=true, Norway: https://www.helsedirektoratet.no/retningslinjer/demens, Scotland: file:///C:/Users/SCHMAC~ 1/AppData/Local/Temp/0117212.pdf, Spain: https://portal.guiasalud.es/wp-content/uploads/2018/12/GPC_484_Alzheimer_AIAQS_comp_eng.pdf, Sweden: https://www.socialstyrelsen.se/regler-och-riktlinjer/nationella-riktlinjer/slutliga-riktlinjer/demens/).

## Appendix

**Table 3:** List of responding organizations

Country	Organizations responding to the e-mail request
Austria	Alzheimer Austria Federal Ministry of Social Affairs, Health, Care and Consumer Protection
Belgium	Vlaamse Regering Agence wallonne pour une vie de qualité (AViQ) Expertisecentrum Dementie Vlaanderen
Bulgaria	Foundation Compassion Alzheimer Bulgaria
Croatia	Alzheimer Croatia Klinika za psihijatriju Vrapče
Cyprus	Ministry of Health
Czech Republic	Ministry of Health of the Czech Republic
Denmark	Danish Ministry of Health Danish Health Authority
Germany	Federal Ministry of Health
England	Department of Health and Social Care
Estonia	Ministry of Social Affairs
Finland	Ministry of Social Affairs and Health
France	French Society of Geriatrics and Gerontology Institut national de la santé et de la recherche médicale (Inserm)
Greece	Hellenic Association of Geriatrics and Gerontology Aristotle University of Thessaloniki
Hungary	National Healthcare Service Center
Ireland	National Dementia Office Department of Health
Italy	Italian Society of Gerontology and Geriatrics
Latvia	Ministry of Health of the Republic of Latvia
Lithuania	Ministry of Health of The Republic of Lithuania
Luxembourg	Ministry of Health
Malta	Malta Dementia Society
Netherlands	Netherlands Centre of Expertise for Long-Term Care (Vilans)
Northern Ireland	Department of Health, Social Services and Public Safety
Poland	Ministry of Health of the Republic of Poland
Portugal	Chronic Diseases Research Center (CEDOC)
Romania	Alzheimer Society Romania
Scotland	Scottish Government
Sweden	Ministry of Health and Social Affairs Svenskt Demenszentrum
Slovakia	German Embassy Bratislava Centrum Memory Bratislava
Slovenia	Slovenian Geriatric Medicine Society
Spain	Ministry of Health, Social Services and Equality
Wales	Department of Health and Social Services
Iceland	Ministry of Health
Liechtenstein	Ministry of Society/Department of Health Demenz Liechtenstein
Norway	Ministry of Health and Care Services
Switzerland	Association Alzheimer Suisse Federal Office of Public Health

**Table 4:** Quoted text excerpts from migration-related guidelines

Country	Document title	Theme	Quoted text excerpt (original language)	English translation
Austria	Medical guideline for integrated care for dementia patients	Dementia diagnosis – Diagnostic tools	“Der Mini-Cog ist ein einfaches Testverfahren zur Früherkennung von Demenzerkrankungen, dessen Aussagekraft durch kulturelle und sprachliche Unterschiede sowie durch unterschiedliche Bildungsniveaus nicht beeinträchtigt wird.”	The Mini-Cog is a simple test procedure for the early detection of dementia diseases, whose significance is not affected by cultural and linguistic differences and different levels of education.
Belgium (Flanders)	You and me, together we are human: a reference framework for quality of life, housing and care for people with dementia	Challenges of diversity for healthcare	“Our Western society is getting more colourful and diverse. This is enriching but it does make some things more challenging for carers.”	
Denmark	National clinical guidelines on the examination and treatment of dementia	Dementia diagnosis – Diagnostic tools	“Der foreligger en dansk validering af RUDAS baseret på testning af 137 patienter (heraf 34 med indvandrerbaggrund) fra demensudredningsenheder.”	There is a Danish validation of RUDAS based on testing of 137 patients (including 34 with immigrant background) from dementia assessment units.
England/Wales	Dementia: Assessment, management and support for people living with dementia and their carers	Dementia diagnosis – Diagnostic tools	“The committee agreed that some tests (e.g., MoCA) are less robust in certain population groups due to cultural differences (educational levels, language issues), and this can skew the resulting diagnosis of dementia/continued suspicion of dementia.”	
Germany	S-3 guideline Dementias	Dementia diagnosis – Diagnostic tools -Recommendations	“Ausführliche neuropsychologische Tests sollten bei fraglicher oder leichtgradiger Demenz zur differenzialdiagnostischen Abklärung eingesetzt werden. […] Beeinflussende Variablen, wie z.B. […] soziokultureller Hintergrund oder Sprachkenntnisse, müssen berücksichtigt werden.”	Extensive neuropsychological tests should be used for differential diagnosis of questionable or mild dementia. [...] Influencing variables, such as […] socio-cultural background or language skills, must be taken into account.
Ireland	Dementia: Diagnosis & Management in General Practice	Dementia diagnosis – Diagnostic tools	1. “The MIS is especially appropriate for use with ethnic minorities, as it does not show educational or language bias.” 2. “The Mini-Cog is less affected by subject ethnicity, language, and education […].”	
Northern Ireland	Dementia: A NICE–SCIE Guideline on supporting people with dementia and their carers in health and social care	Development and effects of Dementia - Vulnerability	“People from minority ethnic groups have special considerations. Increased incidence of hypertension and diabetes among African, Caribbean and Asian people increases the risk of developing vascular dementia in older age.”	
Norway	National professional guidelines on dementia	Dementia diagnosis and Care - Validity and Access - Causes	1. “Utredning av. demens hos personer med minoritetsbakgrunn kan være utfordrende fordi pasienten har en annen kultur- og språkbakgrunn som kan gi kommunikasjonsutfordringer i konsultasjonen.” 2. “Det kan også være et problem at kognitive tester som brukes er så kultur- og språkspesifikke at de ikke er egnet som utredningsverktøy I forskjellige innvandrergrupper.”	1. Investigating dementia in people with minority backgrounds can be challenging because the patient has a different cultural and language background, which can present communication challenges in the consultation. 2. It can also be a problem that the cognitive tests used are so culture and language specific that they are not suitable as an assessment tool in different immigrant groups.
Scotland	Standards of Care for Dementia in Scotland	Dementia diagnosis - Recommendations,	“People worried about their memory have timely access to services for assessment, including those who may be seldom heard, e.g., […] black and ethnic minority communities […].”	
Spain	Clinical Practice Guideline on the Comprehensive Care of People with Alzheimer’s Disease and other Dementias	Care - Impact of Culture and Language	1. “The cognitive assessment tests used have a low positive predictive value and the disadvantage of not being able to many patients due to the limitations of these tests, which may be influenced by the patient’s age, sex, culture and education.” 2. “The risk of abuse is multifactorial, combining personal, family, social and cultural elements.”	
Sweden	Evaluated version of the policy: Health and social care at Dementia	Healthcare services - Availability	“Utvärderingen tyder på att det finns brister i vården och omsorgen för personer från andra länder – både när det gäller möjligheten till tidig upptäckt, adekvat läkemedelsbehandling och vilka insatser som ges när någon har fått en demensdiagnos.”	The evaluation indicates that there are deficiencies in health and social care for people from other countries - both in terms of opportunity for early detection, adequate drug treatment, and what efforts are made when someone has a dementia diagnosis.

## Abbreviations

ADI: Alzheimer Disease International
DGPPN: Deutsche Gesellschaft für Psychiatrie und Psychotherapie, Psychosomatik und Nervenheilkunde
EFTA: European Free Trade Association
MIS: Memory Impairment Screen
MMSE: Mini-Mental State Examination
NAKMI: Nasjonalt kompetansesenter for migrasjons- og minoritetshelse
NDP: National dementia plan
NDPs: National dementia plans
NICE: National Institute for Health and Care Excellence
PGPPN: Society of Psychiatry and Psychotherapy, Psychosomatics and Neurology
PwM: People with a migration background
RUDAS: Rowland Universal Dementia Assessment Scale
SCIE: Social Care Institute for Excellence
